# Supplementary material for: Global irrigation contribution to wheat and maize yield
Source: Nat Commun. 2021 Feb 23;12:1235. doi: 10.1038/s41467-021-21498-5 (PMC7902844; doi:10.1038/s41467-021-21498-5)
Supplement: Supplementary file 1 — Supplementary Information [file 41467_2021_21498_MOESM1_ESM.pdf]

Supplementary Figure 1. Spatial distribution of ΔY from climate analogue approach for **a** wheat and **b** maize over contemporary growing area.

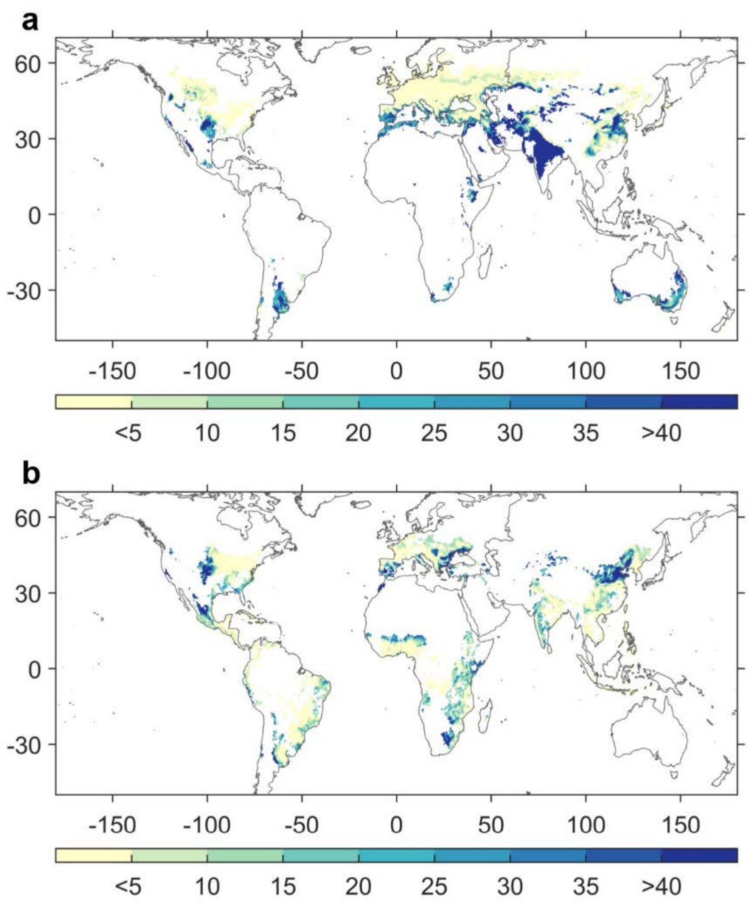

Supplementary Figure 2. Spatial distribution of mean growing season temperature and precipitation for wheat (top panels) and for maize (bottom panels) during 1980-2010. **a** mean growing season temperature (°C) for wheat; **b** mean growing season precipitation (mm) for wheat; **c** mean growing season temperature (°C) for maize; **d** mean growing season precipitation for maize (mm).

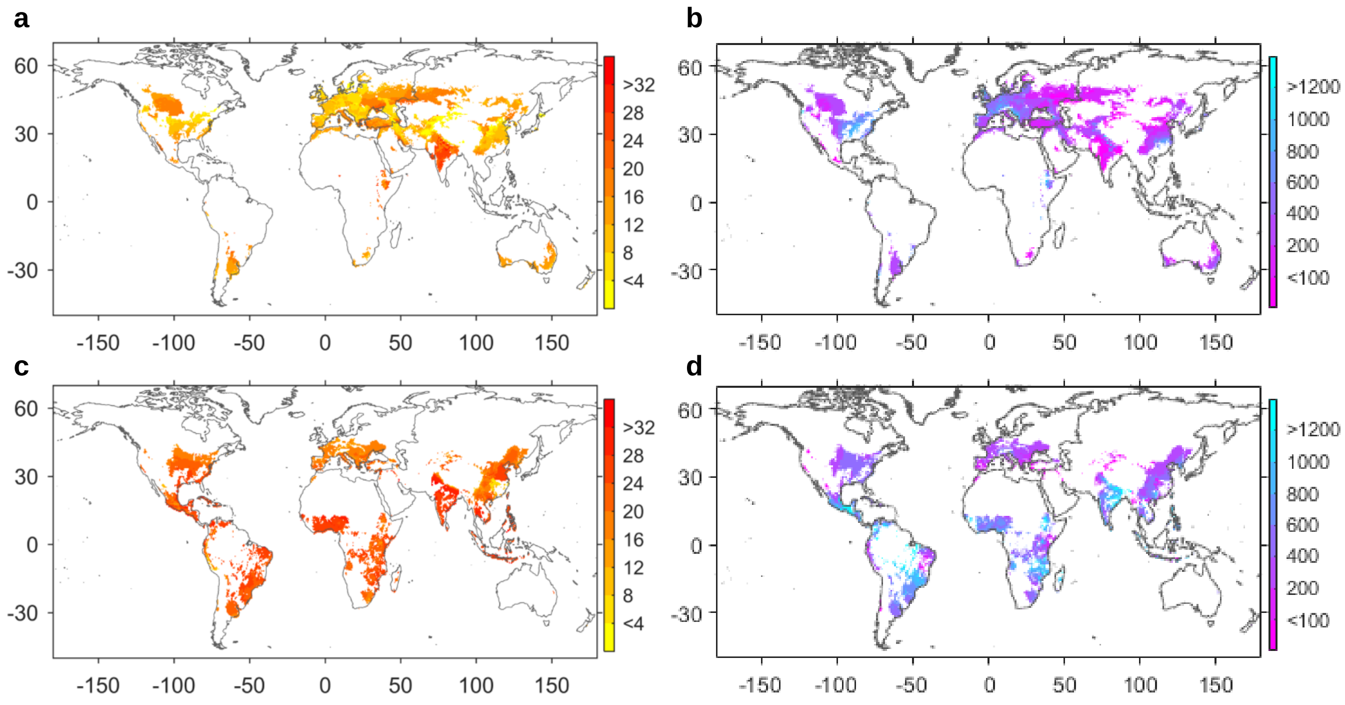

Supplementary Figure 3. Partial correlation in the spatial domain between ΔY and climatic variables (potential evapotranspiration (PET) and mean annual precipitation (MAP)) for wheat (top panel) and for maize (bottom panel). **a,c** bivariate mapping for spatial distribution of the partial correlation coefficient between ΔY and PET ($R_{\Delta Y,PET}$) and that between ΔY and MAP ($R_{\Delta Y,MAP}$). **b,d** Percentage of cropland area where ΔY is controlled by PET or precipitation depending on the chosen threshold (x-axis) for the partial correlation coefficients. PET was calculated following Penman-Monteith equations provided by Harris et al.[1].

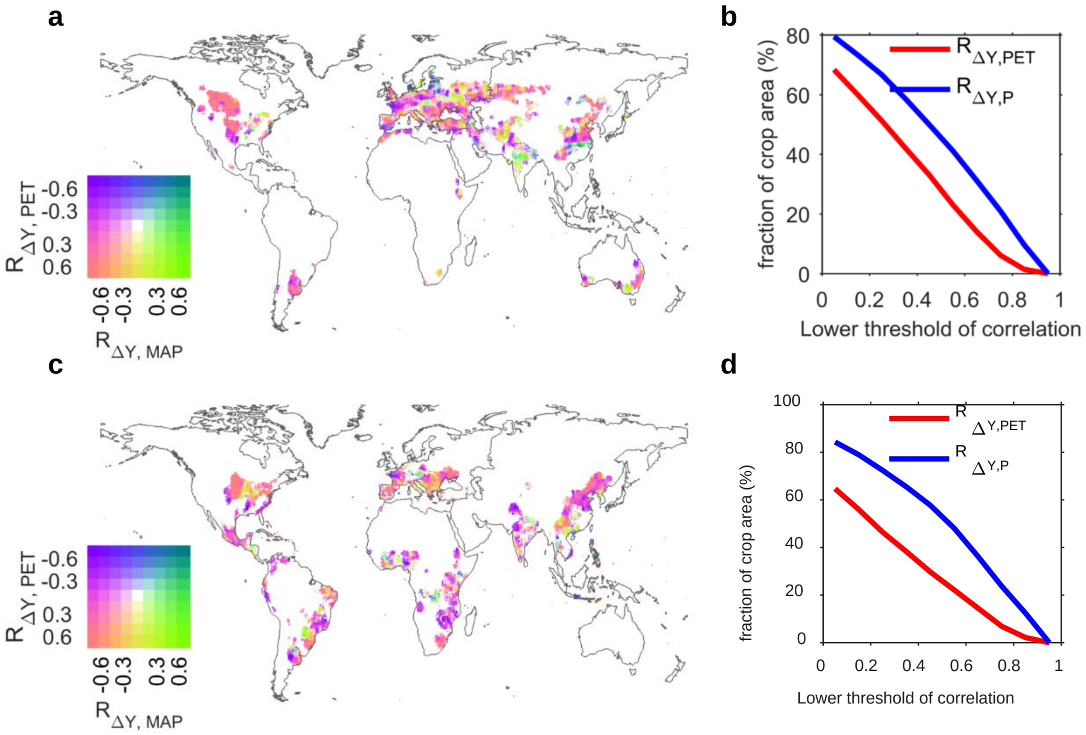

Supplementary Figure 4. Partial correlation in the spatial domain (3.5°×3.5° moving windows) between ΔY and climatic variables (mean annual temperature (MAT) and mean annual precipitation (MAP)) for wheat (top panel) and for maize (bottom panel). **a,c** bivariate mapping for spatial distribution of the partial correlation coefficient between ΔY and MAT ($R_{\Delta Y,MAT}$) and that between ΔY and MAP ($R_{\Delta Y,MAP}$). **b,d** Percentage of cropland area where ΔY is controlled by temperature or precipitation depending on the chosen threshold (x-axis) for the partial correlation coefficients. Same to Figure 3 but using MAT to replace PET.

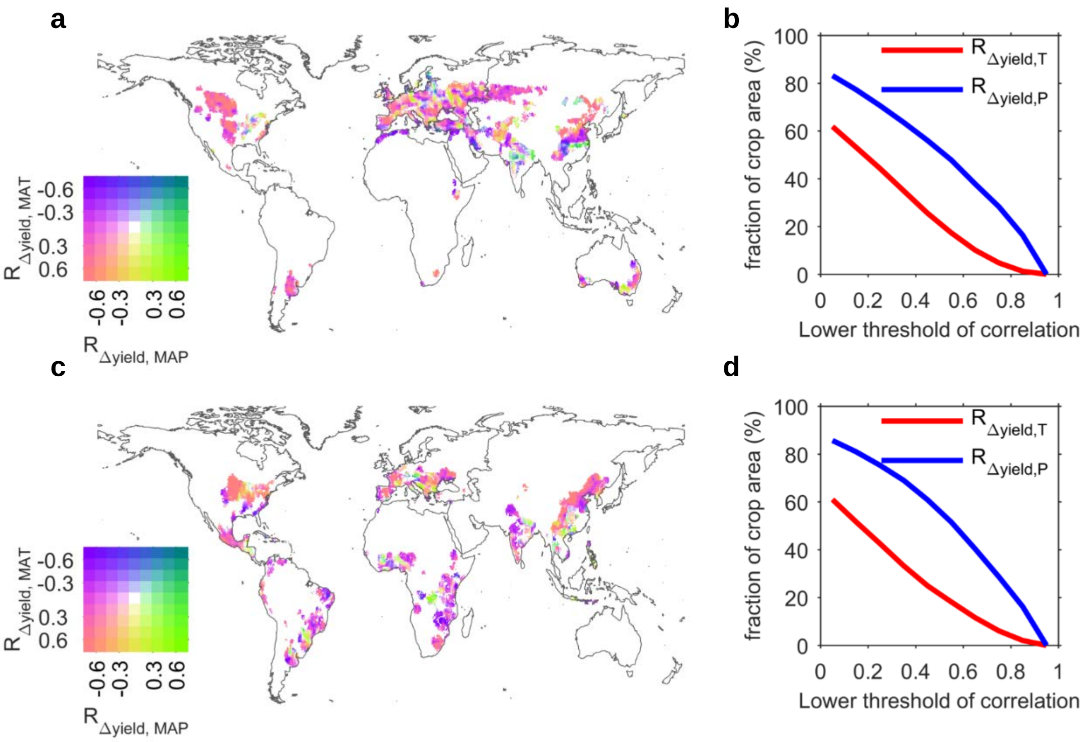

Supplementary Figure 5. The spatial distribution of the difference between irrigation demand and available runoff resources determined with maximum runoff usage threshold of **a** 20% and **b** 40%.

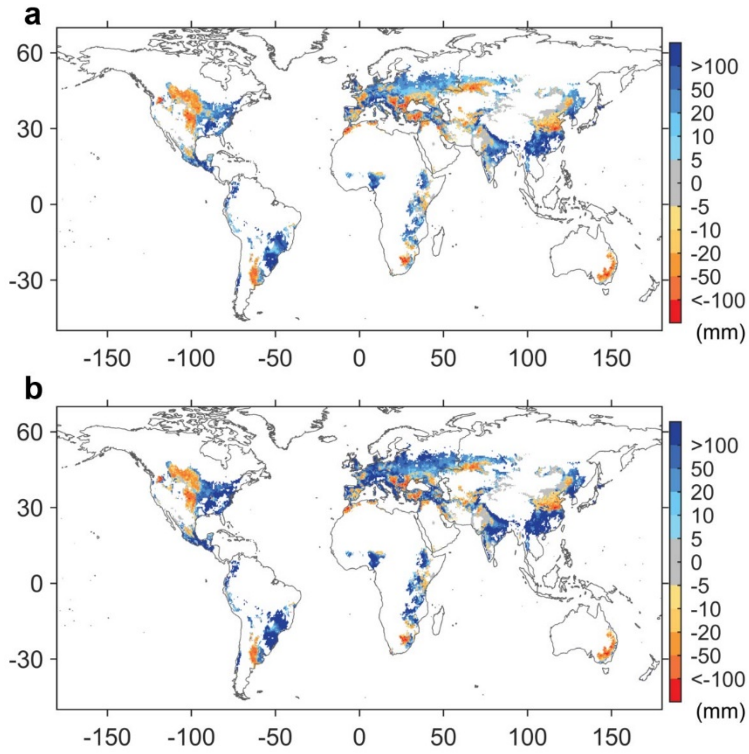

Supplementary Figure 6. Spatial distribution of prevalence of undernourishment (%) during 2000-2010 according to Food and Agriculture Organization of the United Nations (https://unstats.un.org/sdgs/indicators/database/?indicator=2.1.1). Solid black lines delineate major producers of wheat and maize and their names.

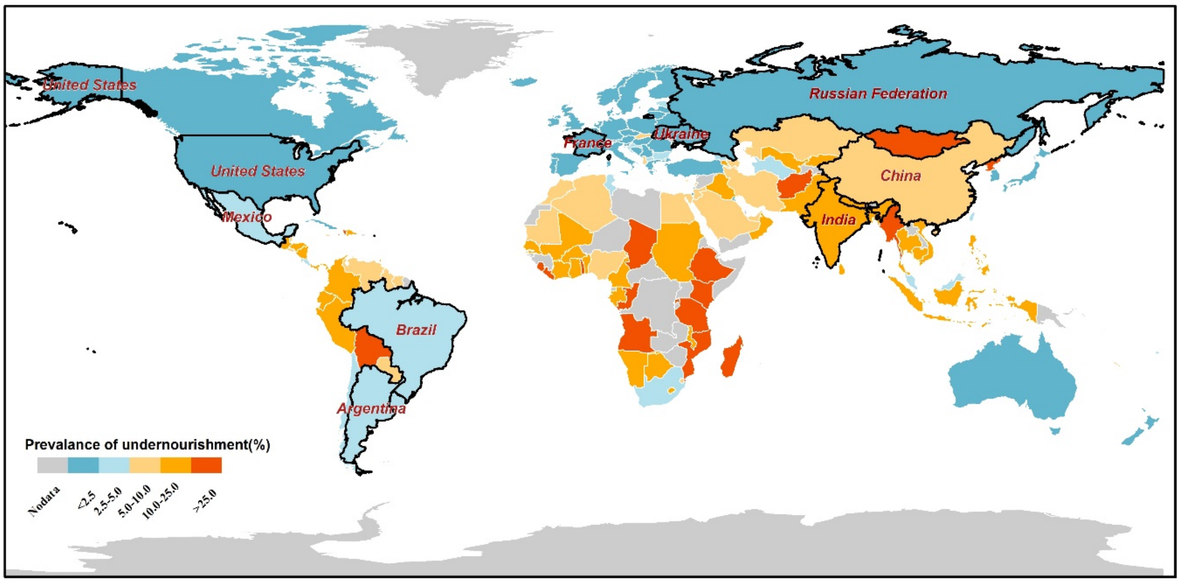

Supplementary Figure 7. Spatial distribution of top 25 river basins having largest rainfed wheat and maize croplands. The color depicts the area of rainfed wheat and maize croplands within the basin according to Portmann et al.[2].

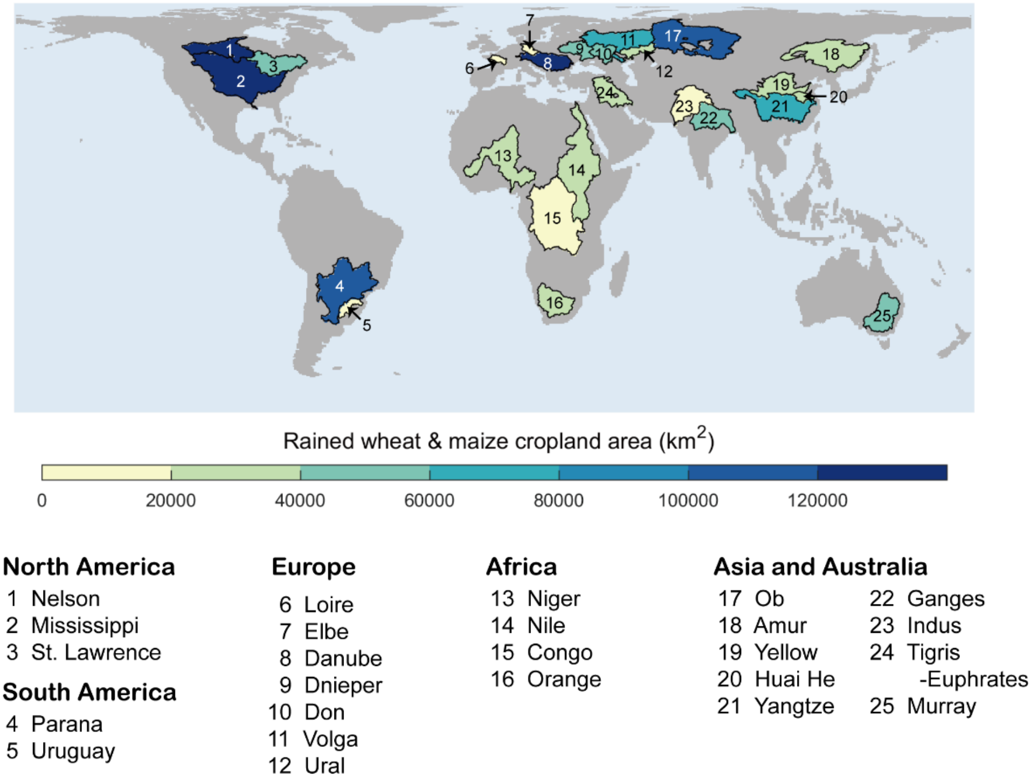

Supplementary Figure 8. Maps of the 10x10 and 20x20 climate zones (bounding the range we utilize) for wheat and maize used by the climate analogue approach.

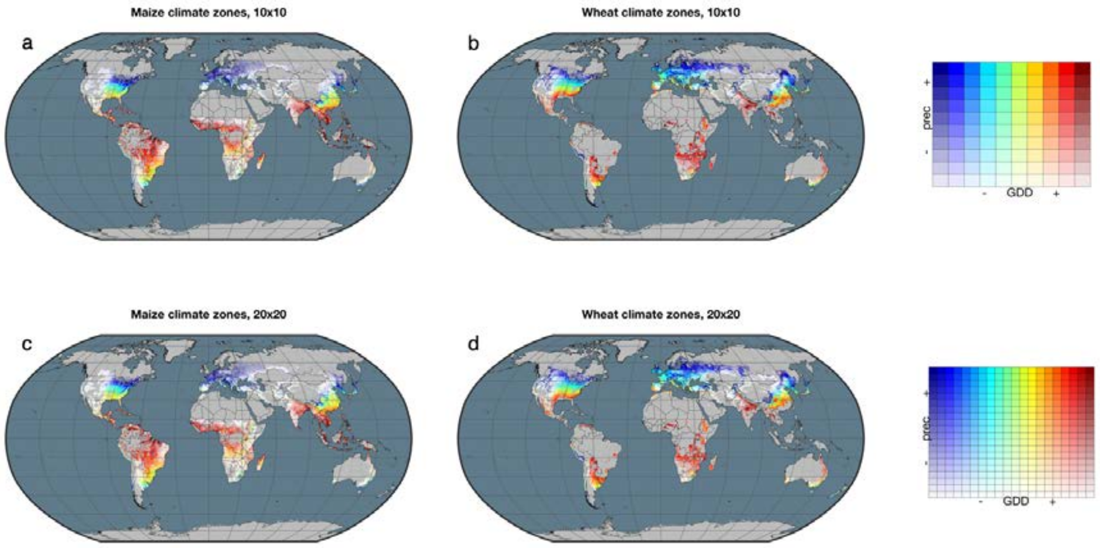

Supplementary Figure 9. The spatial distribution of the standard deviation of ΔY (%) by GGCMs. **a** wheat, **b** maize.

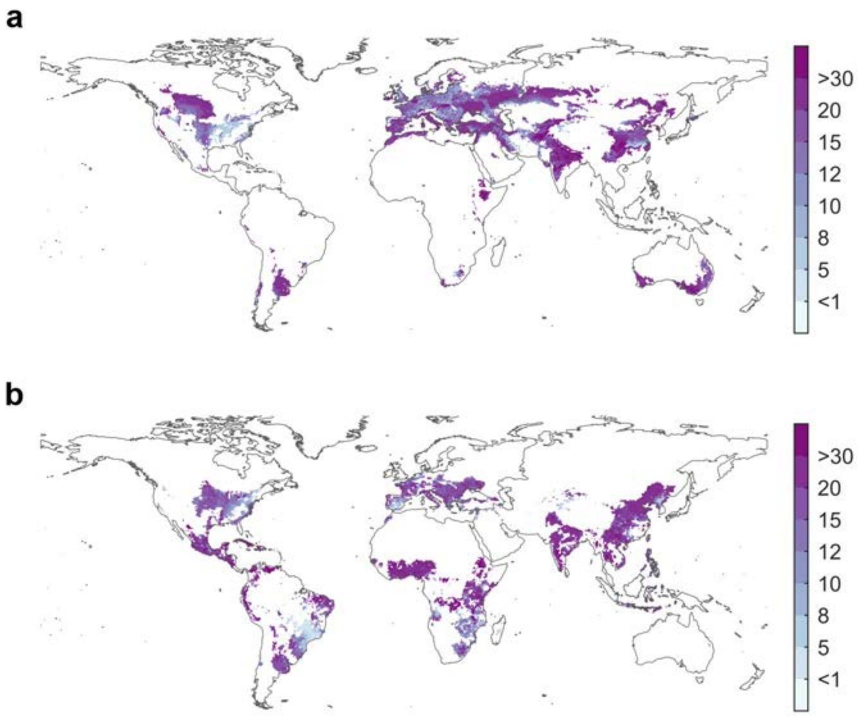

Supplementary Table 1. Balance of river discharge and irrigation demand of contemporary rainfed wheat and maize croplands for 25 river basins with largest rainfed area of wheat and maize. River discharge is the mean annual discharge of the gauging station nearest to the mouth that is represented in GRDC database (https://www.bafg.de/GRDC/EN/01_GRDC/13_dtbse/database_node.html). Irrigation demand is estimated by reanalyzed irrigation demand by GGCMs (see Methods). Rainfed crop area is derived from Portmann et al.[2].

Basin Name	Rainfed area (10^3 km^2)	Discharge (10^3 m^3)	Demand (10^3 m^3)	Demand to supply ratio (%)
Mississippi	367.2	535.0	60.0	11.2
Danube	126.0	202.3	25.0	12.3
Nelson river	120.4	95.5	14.7	15.4
Ob	110.9	400.4	17.2	4.3
Parana	106.5	476.3	10.6	2.2
Volga	64.7	256.7	2.7	1.1
Yangtze river	60.2	899.4	3.7	0.4
Don	56.5	25.5	7.7	30.0
Murray	51.8	6.7	24.9	372.0
Ganges	47.0	379.6	6.4	1.7
Dniepr	46.7	47.1	3.6	7.7
St.lawrence	45.6	268.2	2.0	0.8
Amur	39.4	314.7	4.2	1.3
Huai River	30.4	27.9	7.8	28.0
Tigris & euphrates	29.7	37.6	15.6	41.6
Yellow river	27.5	45.0	9.3	20.8
Orange	27.2	9.0	13.7	152.4

Niger	25.7	159.5	0.3	0.2
Ural	25.3	9.4	5.0	53.4
Nile	21.2	39.5	1.8	4.5
Congo	19.5	1269.3	0.1	0.0
Indus	19.2	91.6	3.6	3.9
Uruguay	19.0	170.5	0.3	0.2
Elbe river	17.8	22.4	1.6	7.1
Loire	17.3	26.4	4.3	16.1

Supplementary Table 2. Characteristics of used crop models

Model	Type[1]	CO2 effects[2]	Stresses[3]	Fertilizer application[4]	Calibration[5]	Calibrated parameters	Reference[6]
**EPIC-BOKU**	Site-based	RUE, TE	W, T, A, N, P, BD, AL	automatic N input (max 200 kg Ha-1 yr-1) PK (national stat. IFA) dynamic application	Site-specific (EPIC 0810)	NA	Ref. 3
**EPIC-IIASA**	Site-based	RUE, TE	W, T, A, N, P, BD, AL	NP (sub-national stat by Balkovič et al. (2013); Mueller et al. (2012)); P timing: rigid; N timing: automatic (based on N stress)	Site-specific and global	F, HIpot (ric, mai) F (others)	Ref. 3
**EPIC-TAMU**	Site-based	RUE, TE	W, T, H, A, N, P, BD, AL	NPK at planting	Site-specific and global	HIpot (maize)	Ref. 4
**GEPIC**	Site-based	RUE, TE	W, T, A, N, P, BD, AL	NP (national stat: FertiSTAT), dynamic application of N, rigid application of P	Site-specific and global	F HIpot (for maize and rice)	Ref. 5
**LPJ-GUESS**	Ecosystem	LF, SC	W, T	NA	Uncalibrated	NA	Ref. 6
**LPJmL**	Ecosystem	LF, SC	W, T	NA	National	LAImax HI αa	Ref. 7
**ORCHIDEE-crop**	Ecosystem	LF, SC	W,T,N	Automatic N input (IFA)	Uncalibrated	-	Ref. 8
**pAPSIM**	Site-based	RUE	W, T, H, A, N	SPAM by You et al. (2014), (1/2 at planting, 1/2 at day 45)	Site-specific (APSIM)	NA	Ref. 9
**pDSSAT**	Site-based	RUE (for wheat, rice,	W, T, H, A, N	SPAM by You et al. (2014), (1/2 at planting, 1/2 at	Site-specific (DSSAT)	NA	Ref. 9

		maize) and LF (for soybean)		day 45)			
**PEGASUS**	Ecosystem	RUE, TE	W, T, H, N, P, K	NPK (national stat. IFA), annual application	Global	β	Ref. 10

Notes: (NA where not applicable)

[1] Site-based: site-base crop model; Ecosystem: global ecosystem model

[2] Elevated CO_2 effects: LF: Leaf-level photosynthesis (via rubisco or quantum-efficiency and leaf-photosynthesis saturation; RUE: Radiation use efficiency; TE: Transpiration efficiency; SC: stomatal conductance

[3] W: water stress; T: temperature stress; H: specific-heat stress; A: oxygen stress; N: nitrogen stress; P: phosphorus stress; K: potassium stress; BD: bulk density; AL: aluminum stress (based on pH and base saturation)

[4] Fertilizer application, timing of application; NPK annual application of total NPK (nutrient-stress factor); source of fertilizer application data; timing: annual or dynamic

[5] F: fertilizer application rate; HIpot: Potential harvest index; LAImax: maximum LAI under unstressed conditions; HI: harvest index; αa: factor for scaling leaf-level photosynthesis to stand level; β: radiation-use efficiency factor; TH: Total Heat unit required for the maturity; TC: Technological coefficient; TS: Temperature sensitivity of photosynthesis; LR: ratio of leaf to above ground biomass.

[6] See Supplementary Reference.

Supplementary Table 3. irrigation contribution to yield (ΔY) (%) for major wheat and maize producers

Wheat		Maize	
China	42.2	USA	24.9
India	53.5	China	22.6
Russia	15.7	Brazil	22.2
USA	31.9	France	24.4

Supplementary Table 4. Web links to GGCMI model output

**Model**	**Wheat**	**Maize**
**EPIC-BOKU**	http://dx.doi.org/10.5281/zenodo.1404761	http://dx.doi.org/10.5281/zenodo.1404767
**EPIC-IIASA**	http://dx.doi.org/10.5281/zenodo.1403195	http://dx.doi.org/10.5281/zenodo.1403203
**EPIC-TAMU**	http://dx.doi.org/10.5281/zenodo.1409013	http://dx.doi.org/10.5281/zenodo.1409009
**GEPIC**	http://dx.doi.org/10.5281/zenodo.1408571	http://dx.doi.org/10.5281/zenodo.1408577
**LPJ-GUESS**	http://dx.doi.org/10.5281/zenodo.1408623	http://dx.doi.org/10.5281/zenodo.1408647
**LPJmL**	http://dx.doi.org/10.5281/zenodo.1403013	http://dx.doi.org/10.5281/zenodo.1403073
**ORCHIDEE-crop**	http://dx.doi.org/10.5281/zenodo.1408191	http://dx.doi.org/10.5281/zenodo.1408199
**pAPSIM**	http://dx.doi.org/10.5281/zenodo.1403183	http://dx.doi.org/10.5281/zenodo.1403189
**pDSSAT**	http://dx.doi.org/10.5281/zenodo.1403171	http://dx.doi.org/10.5281/zenodo.1403181
**PEGASUS**	http://dx.doi.org/10.5281/zenodo.1409546	http://dx.doi.org/10.5281/zenodo.1409550

Supplementary References

1. Harris I, Jones PD, Osborn TJ, Lister DH. Updated high-resolution grids of monthly climatic observations - the CRU TS3.10 Dataset. *International Journal of Climatology* **34**, 623-642 (2014).
2. Portmann FT, Siebert S, Döll P. MIRCA2000-Global monthly irrigated and rainfed crop areas around the year 2000: A new high-resolution data set for agricultural and hydrological modeling. *Global Biogeochem. Cycles*. **24**, GB1011 (2010).
3. Izaurralde RC, Williams JR, McGill WB, Rosenberg NJ, Jakas MCQ. Simulating soil C dynamics with EPIC: Model description and testing against long-term data. *Ecological Modelling* **192**, 362-384 (2006).
4. Liebig M, Franzluebbers AJ, Follett RF. Managing agricultural greenhouse gases: Coordinated agricultural research through GRACEnet to address our changing climate. Academic Press (2012).
5. Liu JG, Williams JR, Zehnder AJB, Yang H. GEPIC - modelling wheat yield and crop water productivity with high resolution on a global scale. *Agricultural Systems* **94**, 478-493 (2007).
6. Lindeskog M, et al. Implications of accounting for land use in simulations of ecosystem carbon cycling in Africa. *Earth Syst Dyn* **4**, 385-407 (2013).
7. Waha K, van Bussel LGJ, Muller C, Bondeau A. Climate-driven simulation of global crop sowing dates. *Global Ecology and Biogeography* **21**, 247-259 (2012).
8. Wang X. Impacts of environmental change on rice ecosystems in China: development, optimization and application of ORCHIDEE-crop model.Peking University (2015).
9. Elliott J, et al. The parallel system for integrating impact models and sectors (pSIMS). *Environ. Modell. Softw.* **62**, 509-516 (2014).
10. Deryng D, Conway D, Ramankutty N, Price J, Warren R. Global crop yield response to

extreme heat stress under multiple climate change futures. *Environmental Research Letters* **9**,034011 (2014).
