# Supplementary material for: Global irrigation contribution to wheat and maize yield
Source: Nat Commun. 2021 Feb 23;12:1235. doi: 10.1038/s41467-021-21498-5 (PMC7902844; doi:10.1038/s41467-021-21498-5)
Supplement: Supplementary file 3 — Reporting Summary [file 41467_2021_21498_MOESM3_ESM.pdf]

nature research

Corresponding author(s): Xuhui Wang
Last updated by author(s): Jan 15, 2021

Reporting Summary

Nature Research wishes to improve the reproducibility of the work that we publish. This form provides structure for consistency and transparency in reporting. For further information on Nature Research policies, seeAuthors & Referees and theEditorial Policy Checklist .

Statistics

For all statistical analyses, confirm that the following items are present in the figure legend, table legend, main text, or Methods section.

n/a	Confirmed	
☐	☒	The exact sample size (n) for each experimental group/condition, given as a discrete number and unit of measurement
☐	☒	A statement on whether measurements were taken from distinct samples or whether the same sample was measured repeatedly
☐	☒	The statistical test(s) used AND whether they are one- or two-sided<br>*Only common tests should be described solely by name; describe more complex techniques in the Methods section.*
☐	☒	A description of all covariates tested
☐	☒	A description of any assumptions or corrections, such as tests of normality and adjustment for multiple comparisons
☐	☒	A full description of the statistical parameters including central tendency (e.g. means) or other basic estimates (e.g. regression coefficient) AND variation (e.g. standard deviation) or associated estimates of uncertainty (e.g. confidence intervals)
☐	☒	For null hypothesis testing, the test statistic (e.g. F, t, r) with confidence intervals, effect sizes, degrees of freedom and P value noted<br>*Give P values as exact values whenever suitable.*
☐	☒	For Bayesian analysis, information on the choice of priors and Markov chain Monte Carlo settings
☒	☐	For hierarchical and complex designs, identification of the appropriate level for tests and full reporting of outcomes
☐	☒	Estimates of effect sizes (e.g. Cohen's d, Pearson's r), indicating how they were calculated

Our web collection on statistics for biologists contains articles on many of the points above.

Software and code

Policy information about availability of computer code

Data collection	no software was used
Data analysis	Our analyses were performed using MATLAB (R2017b). Details were reported in the Methods section.

For manuscripts utilizing custom algorithms or software that are central to the research but not yet described in published literature, software must be made available to editors/reviewers. We strongly encourage code deposition in a community repository (e.g. GitHub). See the Nature Research guidelines for submitting code & software for further information.

Data

Policy information about availability of data

All manuscripts must include a data availability statement. This statement should provide the following information, where applicable:

- Accession codes, unique identifiers, or web links for publicly available datasets
- A list of figures that have associated raw data
- A description of any restrictions on data availability

GGCMI model data can be accessed under open data license CC BY 4.0 in https://zenodo.org. (Supplementary Table 4). Attainable irrigated and rainfed yield by Climate Analog approach can be accessed upon request to Prof. N Mueller (nathan.mueller@rams.colostate.edu). Global gridded river discharge can be downloaded from http://www.grdc.sr.unh.edu/.

Field-specific reporting

Please select the one below that is the best fit for your research. If you are not sure, read the appropriate sections before making your selection.

Ecological, evolutionary & environmental sciences study design

All studies must disclose on these points even when the disclosure is negative.

Study description	We applied a Bayesian framework to provide a reanalysis of global irrigation contribution to wheat and maize yield based on 10 global gridded crop models and empirical estimates by Climate Analog approach. The reanalyzed difference between attainable rainfed and irrigated yield was validated against statistical surveys. The reanalyzed irrigation demands were contested against available river discharge, identifying regional differences in the potential to applying irrigation to achieve higher yields.
Research sample	crop yield from simulations by ten global gridded crop models (EPIC-BOKU, EPIC-IIASA, EPIC-TAMU, GEPIC, LPJ-GUESS, LPJmL, ORCHIDEE-crop, pAPSIM, pDSSAT and PEGASUS), Climate Analog dataset and gridded USDA dataset; The global river discharge from UNH-GRDC dataset.
Sampling strategy	We used all available samples.
Data collection	The authors performed global crop model simulations following the phase 1 protocol of GGCMI. An updated version of Climate Analog dataset published by Mueller et al. (2012) was performed and provided by Dr. N Mueller. Global gridded river discharge was downloaded from http://www.grdc.sr.unh.edu/. Updated version of USDA gridded dataset (Chauberger et al., 2019) was provided by Dr. J Elliot.
Timing and spatial scale	1980-2010, global.
Data exclusions	No data was excluded.
Reproducibility	Not relevant as we used all possible data globally.
Randomization	Not relevant as all the contemporary wheat and maize growing area was studied.
Blinding	Global crop modelers do not have access to climate analog dataset before submitting the simulations.

Did the study involve field work? ☐ Yes ☒ No

Reporting for specific materials, systems and methods

We require information from authors about some types of materials, experimental systems and methods used in many studies. Here, indicate whether each material, system or method listed is relevant to your study. If you are not sure if a list item applies to your research, read the appropriate section before selecting a response.

Materials & experimental systems

n/a	Involved in the study
☒	☐ Antibodies
☒	☐ Eukaryotic cell lines
☒	☐ Palaeontology
☒	☐ Animals and other organisms
☒	☐ Human research participants
☒	☐ Clinical data

Methods

n/a	Involved in the study
☒	☐ ChIP-seq
☒	☐ Flow cytometry
☒	☐ MRI-based neuroimaging
